# Antimicrobial Resistance Genes in Clinical *Escherichia coli* Strains from Livestock and Poultry in Shandong Province, China During 2015–2020

**DOI:** 10.3390/antibiotics14010095

**Published:** 2025-01-15

**Authors:** Miaoli Wang, Shaopeng Wu, Yao Wang, Feng Chen, Zhangqi Shen, Zouran Lan

**Affiliations:** 1 National Key Laboratory of Veterinary Public Health and Safety, College of Veterinary Medicine, China Agricultural University, Beijing 100193, China; miaoliwangchina@126.com; 2 Shandong Center for Animal Disease Control and Prevention, Shandong Centre for Zoonotic Disease Surveillance, Jinan 250100, China; wenshuowy@163.com (Y.W.); chenfengvet@126.com (F.C.); 3 Shandong Provincial Key Laboratory of Zoonoses, College of Veterinary Medicine, Shandong Agricultural University, Tai’an 271018, China; wsp@sdau.edu.cn

**Keywords:** MDR *E. coli*, ARGs, livestock and poultry, correlation analysis

## Abstract

Antimicrobial resistant (AMR) *Escherichia coli* (*E. coli*) isolated from animals may lead to antibiotic treatment failure and economic losses to farmers. The co-existence of antimicrobial resistant genes (ARGs) in the same isolate presents a major challenge for the prevention and control of infection in multidrug-resistant (MDR) Gram-negative organisms. There have been a lot of studies on the antibiotic resistance of *E. coli* in livestock and poultry, but few of them have focused on clinical pathogens. **Objective:** The aim of this study was to explore the genetic characteristics, co-occurrence, and correlations between ARGs of *E. coli* isolated from the pathological tissues of livestock and poultry in Shandong Province, East China during 2015–2020. **Methods:** A total of 158 *E. coli* strains were collected and subjected to antimicrobial susceptibility testing and sequencing by whole-genome Next Generation Sequencing (NGS). **Results:** MDR strains accounted for 46.20% of the 158 *E. coli* strains with the highest resistant rate of ciprofloxacin (71.52%). In addition, strains with *bla_NDM-5_/mcr-1.1* and *mcr-1.1/mcr-3.24* were found in chickens, while three strains with *Tet(X4)* were found in pigs. In addition, the most common serotypes detected were the O serotype (76/158) and H serotype (36/158). Moreover, seventy-one STs were found and the most common STs were ST10 (6.33%), ST155 (6.33%), and ST101 (5.69%). The genetic environment analysis of the phylogroups revealed that *E. coli* belonging to phylogroup B1, phylogroup A, and phylogroup C constituted 39.87%, 27.85%, and 15.19%, respectively. Through the correlation analysis, *mcr* genes were observed to have certain relationships with ARGS such as *bla_TEM_*, *floR*, *catA/B*, and *oqx*. **Conclusions:** This study demonstrates the high prevalence and gene diversity of MDR *E. coli* isolated from a clinic in Shandong Province, East China. We predicted the transmission risk of animal-borne *Tet(X4)*-bearing and *mcr*-harboring *E. coli* to public health and provided insight into the relationship of co-existence or co-transfer between *mcr* with ARGS. These relationships present a great challenge for the infection control of MDR Gram-negative organisms.

## 1. Introduction

*Escherichia coli (E. coli)* is a bacterium that naturally resides in the intestines of humans and animals. It is also an opportunistic pathogen and a significant cause of high-cost infections in poultry [1]. Therefore, previous studies have primarily focused on the pathogenicity of clinical *E. coli* rather than on its antimicrobial resistance. However, farmers generally use a large quantity of antibiotics in livestock and poultry breeding to prevent and control infections caused by *E. coli*, which leads to an increase in antibiotic resistance, which can compromise the effectiveness of antibiotics and indirectly promote bacterial invasion. However, people tend to pay more attention to the health threat caused by clinical antibiotic-resistant *E. coli* strains in human medicine, while ignoring the public health risks associated with animal-origin isolates. In the context of ‘One-Health’, the safety of animal production is closely tied to human health. The transmission of antibiotic-resistant and pathogenic *E. coli* between different hosts is facilitated by the chain that links animal breeding to human dining tables. Many studies have shown significant similarities between the *E. coli* from animal and human origin [2,3,4,5].

As we all know, *E. coli* is a reservoir that accumulates antibiotic resistance genes and gradually develops into MDR or even pan-drug-resistant (PDR) pathogens. In recent years, several antimicrobial resistance genes, including extended-spectrum-beta-lactamase (ESBL) genes, carbapenemase genes (*bla_NDM_*, *bla_KPC_*), *mcr*, and *tet(X)*, which are generally considered to be quite important, have been detected in *E. coli*. The previous data showed that CTX-M is the most prevalent type in ESBL-producing *E. coli* [6,7]. In particular, MDR CTX-M-15-producing ST131 *E. coli* clones have spread worldwide. It is reported that *E. coli* is one of the major vectors of *bla_NDM_*, and NDM-producing *E. coli* strains have become a challenging public health threat. Faced with the pressure of treating carbapenem-resistant Enterobacteriaceae (CRE), clinicians have to use polymyxin and tigecycline in clinical practice to achieve successful treatment. However, frequent exposure and the increasing use of polymyxin and tetracyclines in clinical and veterinary settings may be a driving factor in the development of polymyxin and tigecycline resistance [7,8]. In *E. coli*, the plasmid-mediated phosphoethanolamine transferase mobile colistin resistance (MCR) is the main factor mediating resistance to polymyxin. Moreover, *E. coli* is one of the dominant bacterial hosts of *mcr-1*. In addition, plasmid-encoded transferable resistance genes *tet(X3)* and *tet(X4)* have been found in *E. coli* in recent years, confirming the high-level resistance to tigecycline.

Shandong Province is one of the most developed provinces of livestock and poultry breeding in China, and the safety of livestock and poultry production is closely related to national health. Previous studies in this region mainly focused on the antibiotic resistance of *E. coli* isolated from healthy livestock and poultry rather than clinical *E. coli* isolates [9,10]. Therefore, to explore the antibiotic resistance characteristics of clinical *E. coli* from livestock and poultry in Shandong Province, we collected *E. coli* isolated from clinical liver or brain samples from livestock and poultry farms in Shandong Province during 2015–2020 and conducted a related study on the prevalence and genetic characteristics of MDR *E. coli*, hoping to provide data support for monitoring the safe production of livestock and poultry.

## 2. Results

### 2.1. Prevalence and Characteristics of the Resistant Phenotype of E. coli Strains

A total of 158 non-duplicate *E. coli* strains were collected, which were isolated and identified from the liver or brain of veterinary hospital cases. A total of 75 and 83 *E. coli* strains were isolated from livestock farms and poultry farms, respectively.

**Figure 1:**
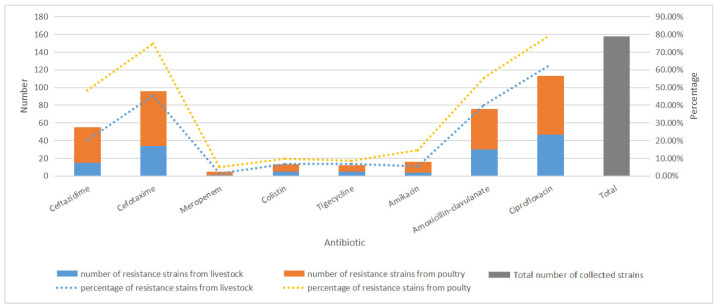
Number and rate trends of *E. coli* strains resistant to antibiotics from livestock and poultry in Shandong Province, China during 2015–2020.

Of the 158 strains, the resistance rates of *E. coli* from poultry to eight drugs were higher than those from livestock (Figure 1). The prevalence of non-resistant (sensitive to all tested antibiotics) strains was 10.13% (16/158); however, 47.47% (75/158) of isolates exhibited multidrug resistance (resistance to more than three drugs) phenotypes. The resistance rates of ciprofloxacin-, β-lactams-, amoxicillin-clavulanate-, and ceftazidime-resistant *E. coli* isolates were 71.52% (113/158), 60.76% (76/158), 48.10% (76/158), and 34.81% (55/158), respectively.

**Figure 2:**
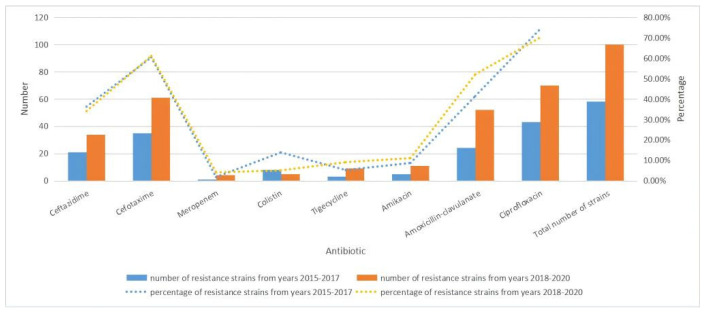
Number and rate trends of *E. coli* strains resistant to antibiotics from 2015 to 2017 and 2018–2020.

In general, 58 *E. coli* strains were isolated from 2015 to 2017 and 100 *E. coli* strains were isolated from 2018 to 2020. By comparing years 2015–2017 and years 2018–2020, the prevalence of β-lactams-, NDM (New Delhi metallo-β-lactamase)-, colistin-, tigecycline-, ceftazidime-, amikacin-, amoxicillin-clavulanate-, and ciprofloxacin-resistant *E. coli* isolates were 60.34% (35/58) and 61.00% (61/100), 1.72% (1/58) and 4.00% (4/100), 13.79% (8/58) and 5% (5/100), 5.17% (3/58) and 9.00% (9/100), 36.21% (21/58) and 34% (34/100), 8.62% (5/58) and 11.00% (11/100), 41.38% (24/58) and 52.00% (52/100), and 74.14% (43/58) and 70.00% (70/100), respectively. Finally, the resistance rates of *E. coli* strains to meropenem, tigecycline, amikacin, and amoxicillin-clavulanate increased during 2018–2020 compared with 2015–2017, while resistance rates to ceftazidime and colistin decreased (Figure 2).

### 2.2. Resistance Genes of E. coli Strains

**Figure 3:**
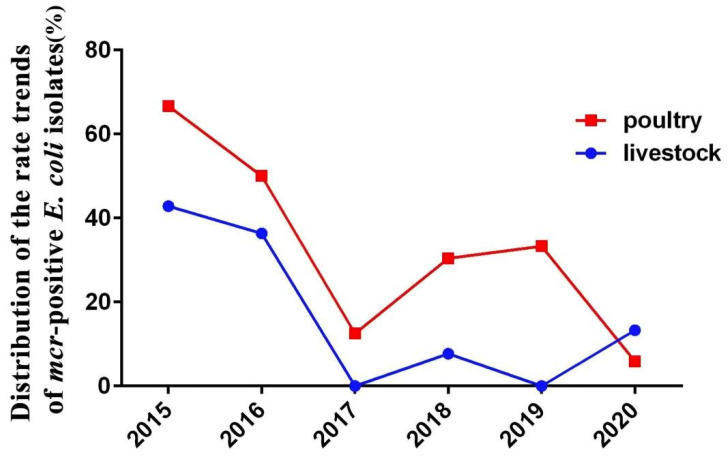
Distribution of the rate trends of *mcr*-harboring *E. coli* strains (n = 37) during years 2015–2017 and 2018–2020.

A total of 158 *E. coli* strains were analyzed for resistant genes in the ResFinder database, and the results are shown in Figure 3. The ratio of isolates harboring genes encoding *Beta-lactam* enzymes was 91.77% (145/158), including *bla_CTX-M_* (50.63%; 80/158), *bla_TEM_* (69.62%; 110/158), *bla_OXA_* (19.62%; 31/158), *bla_CMY-2_* (3.16%; 5/158), and single isolate-harboring *bla_SHV-12_* and *bla_VEB-1_*. *bla_NDM-5_* were found in 2.53% (4/158) of strains from chickens and co-existed with *bla_TEM_*_-1A_ and *bla_TEM_*_-1B_. *mcr-1.1* and *mcr-3.24* genes were found in 23.42% (37/158) of strains from poultry and livestock. *mcr-1.1* and *mcr-3.24* genes were identified with a prevalence of 36.20% (21/58) in years 2015–2017 and 16% (16/100) in years 2018–2020. Among the 37 *mcr*-harboring *E. coli* strains, only 12 stains were phenotypically resistant. Notably, *tet(X)* variants (*Tet(X4)*) were found in 1.90% (3/158) of strains from pigs in 2020; *bla_NDM-5_* and *mcr-1.1* co-existed in two *bla_TEM_*_-1B_-producing *E. coli* strains isolated from chickens in 2019; and *mcr-1.1* and *mcr-3.24* co-existed in two *E. coli* strains isolated from chickens in 2016. Overall, the proportion of *mcr* harboring *E. coli* strains decreased during years 2018–2020 compared with years 2015–2017 (Figure 3).

### 2.3. Serotype and Genetic Molecular ENVIRONMENT Analysis of 158 E. coli Strains

To characterize the molecular profile of these 158 clinical *E. coli* strains, the genome information was analyzed to generate the serotypes, ST, and phylogroups of *E. coli* strains.

The genetic environment analysis of the serotypes revealed that 76 kinds of O serotypes were found in 130 strains, while O8 (8.23%; 13/158) and O78 (5.06%; 8/158) were the most common. Moreover, 39 kinds of H serotypes were found in 154 strains, with H4 (8.86%; 14/158), H9 (8.86%; 14/158), and H21 (8.86%; 14/158) being the most common.

**Figure 4:**
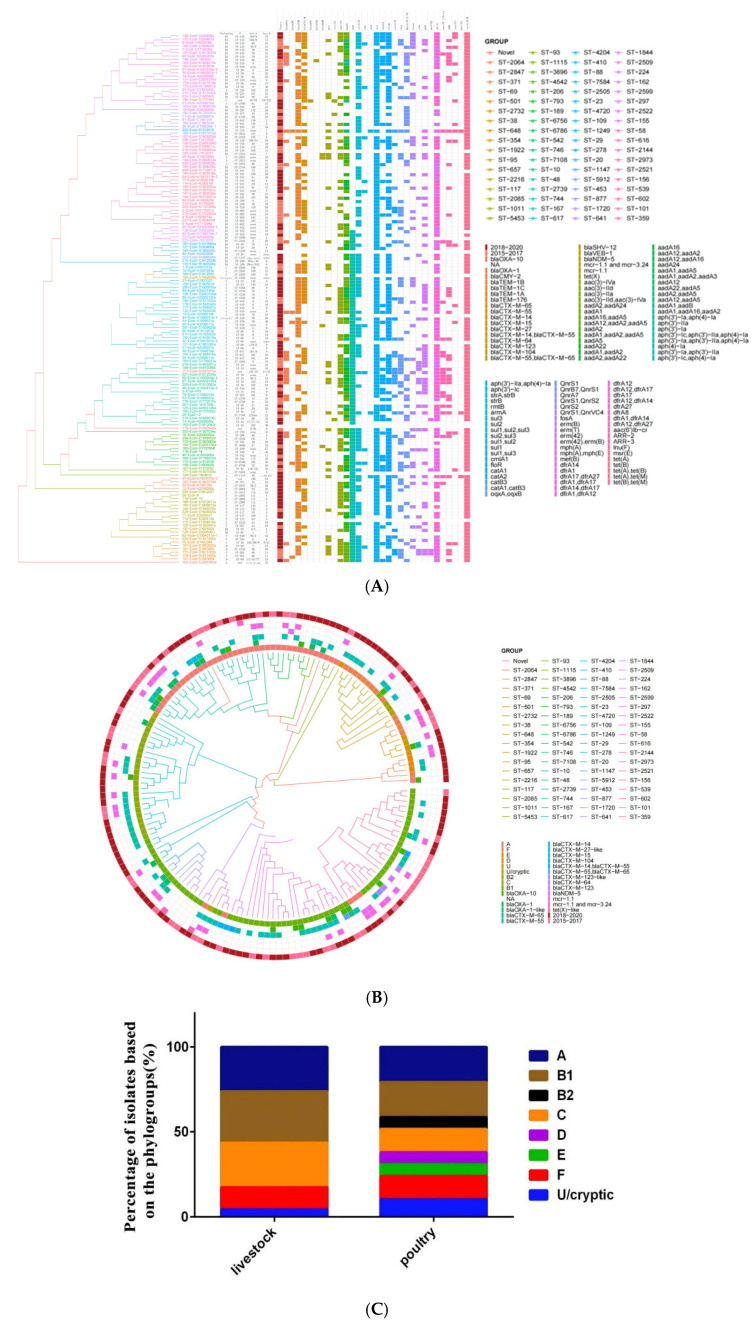
Genetic environment analysis of 158 *E. coli* stains. (**A**) CSI Phylogenetic tree and heatmap based on years, STs, serotypes, and resistance genes of 158 *E. coli* strains. (**B**) Phylogenetic analysis of 158 *E. coli* strains, inferred using the MSTree V2 algorithm based on the cgMLST V1 + Hierarchical Clustering (HierCC) V1 scheme from EnteroBase, colored according to MLSTs, phylogroups terms (A, B1, B2, C, D, E, F, U, cryptic), and resistance genes (*bla_OXA_*, *bla_CTX-M_*, *bla_NDM_*, *mcr*, and *tet(X)*). (**C**) Percentage of isolates (*n* = 158) based on phylogroups from livestock and poultry.

**Figure 5:**
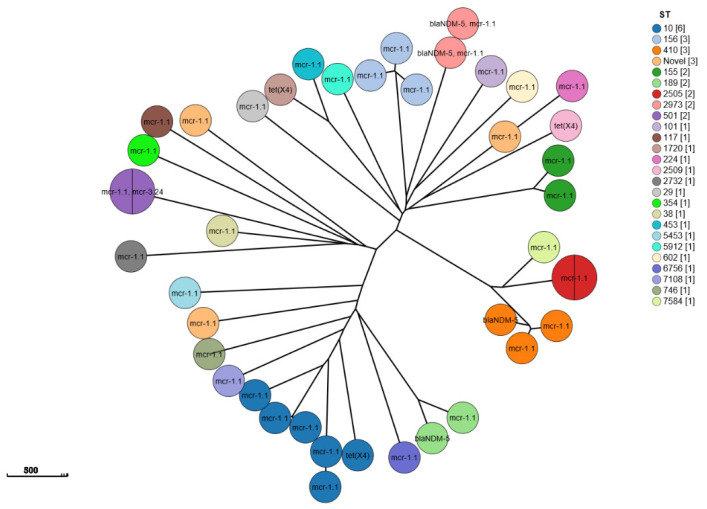
GrapeTree of 42 *E. coli* strains with super-resistant genes (*mcr*, *bla_NDM_*, and *tetX*), inferred using the MSTree V2 algorithm based on the cgMLST V1 + Hierarchical Clustering (HierCC) V1 scheme from EnteroBase, colored according to MLST and labeled according to super-resistant genes. scale bar = 500 µm.

The CSI phylogenetic tree and heatmap analysis of 158 *E. coli* strains showed that phylogroups, STs, and serotypes were related to phylogenetic clusters but resistance genes were not (Figure 4A and Figure 5). The genetic environment analysis of MLST revealed that 71 STs were found in 153 strains with ST10 (6.33%; 10/158), ST155 (6.33%; 10/158), and ST101 (5.69%; 9/158) being the most common, while 5 novel STs were detected (Figure 4B).

The genetic environment analysis of the phylogroups revealed that eight phylogroup terms (A, B1, B2, C, D, E, F, U, cryptic) were found in 158 strains with phylogroup B1 (39.87%; 63/158) and phylogroup A (27.85%; 44/158) being the most common, followed by phylogroup C (15.19%; 24/158), phylogroup F (8.86%; 14/158), phylogroup D (2.53%; 4/158), phylogroup E (1.90%; 3/158), phylogroup B2 (1.27%; 2/158), phylogroup U (1.27%; 2/158), and phylogroup U/cryptic (1.27%; 2/158). Five of these types were found in poultry isolates and eight types in livestock isolates. (Figure 4C).

### 2.4. Serotype and Molecular Typing Analysis of 42 E. coli Strains Carrying bla_NDM_, mcr, and tetX Genes

In order to understand the diversity of the molecular types of these 42 *E. coli* strains carrying *NDM*, *mcr*, and *tetX* genes, the serotypes and MLSTs were analyzed.

**Figure 6:**
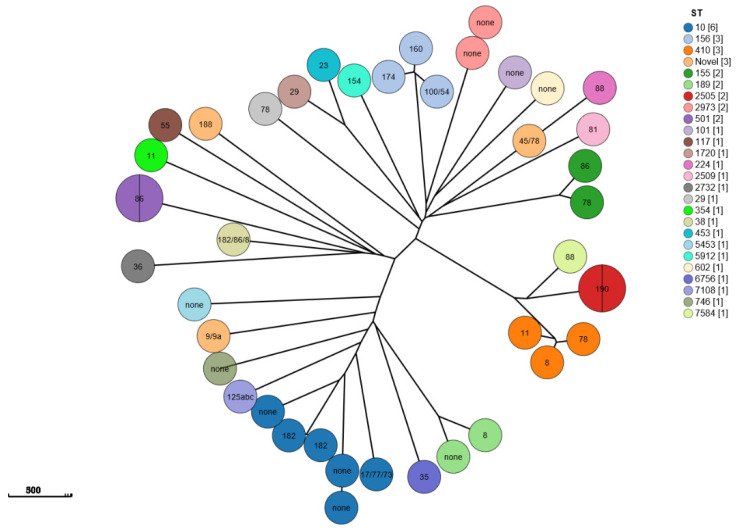
GrapeTree of 42 *E. coli* strains with super-resistant genes (*mcr*, *bla_NDM_* and *tetX*), inferred using the MSTree V2 algorithm based on the cgMLST V1 + Hierarchical Clustering (HierCC) V1 scheme from EnteroBase, colored according to MLST and labeled according to Serotype O. scale bar = 500 µm.

**Figure 7:**
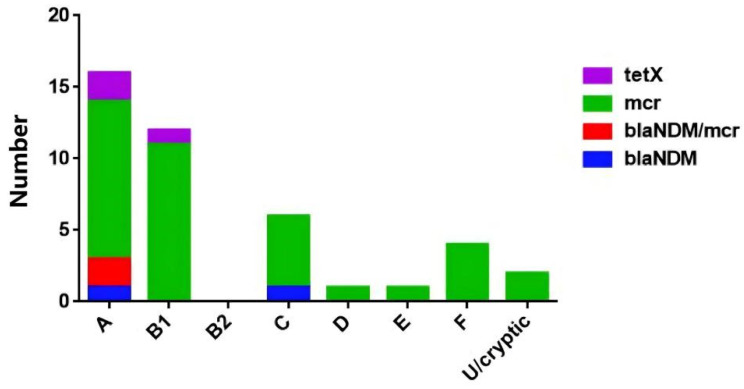
Distribution of 42 *E. coli* strains with genes *mcr*, *bla_NDM_*, and *tetX* based on phylogroups.

In the 37 *E. coli* strains carrying *mcr* genes, the most prevalent MLST was ST-10 (13.51%; 5/37) (Figure 5); 21 O serotypes were found in 28 *E. coli* strains and serotype O was not detected in the other 9 *E. coli* strains (Figure 6). Notably, four phylogroup F strains were found, two of which were *mcr-1.1*/*mcr-3.24*-coharboring strains (Figure 7).

In the four *E. coli* strains carrying *bla_NDM_* genes, the MLSTs were ST-189 (1 strain), ST-410 (1 strain), and ST-2973 (2 strains), respectively (Figure 5); O11:H9 was found in one isolate with *bla_NDM-5_*, and serotype O was not detected in the other three *E. coli* strains. H27, H9, and H16 (2 strains) were found in the four *E. coli* strains (Figure 5 and Figure 6).

In the three *E. coli* strains carrying *tet(X4)* genes, the MLSTs were ST-1720, ST-10, and ST-2509, respectively, and the serotypes were O29:H10, O17 (77 or 73):H9, and O81:H16, respectively, (Figure 5 and Figure 6).

**Figure 8:**
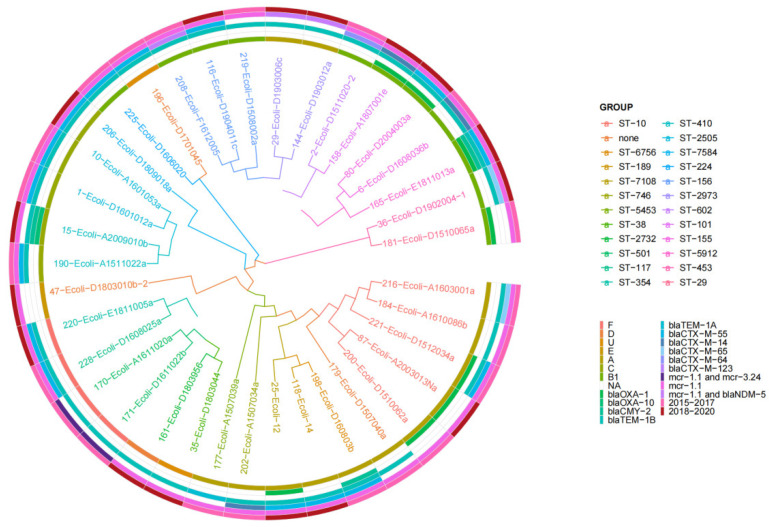
Phylogenetic analysis of the 37 *mcr*-positive (*mcr*-positive and *mcr*+*bla_NDM_* positive) *E. coli* strains.

The phylogenetic analysis of GrapeTree showed that the *mcr*-positive strains were divided into three clusters, *bla_NDM_*-positive strains were distributed into two clusters, and *tet(X)*-positive strains were divided into two clusters, which were in accordance with the cgMLST (Figure 8).

### 2.5. Genetic Background Analysis of mcr-1.1/3.24-Coharboring E. coli Strains

To understand the genetic background of *mcr-1.1*/3.24-coharboring *E. coli* strains, two *E. coli* strains (170-Ecoli-A1611020a and 171-Ecoli-D1611022b) coharboring *mcr-1.1* and *mcr-3.24* genes were selected for long-read complete sequencing. The sequencing result of 170-Ecoli-A1611020a is the same as that of 171-Ecoli-D1611022b with ST 501; therefore, we choose 170-Ecoli-A1611020a to illustrate the genetic background.

**Figure 9:**
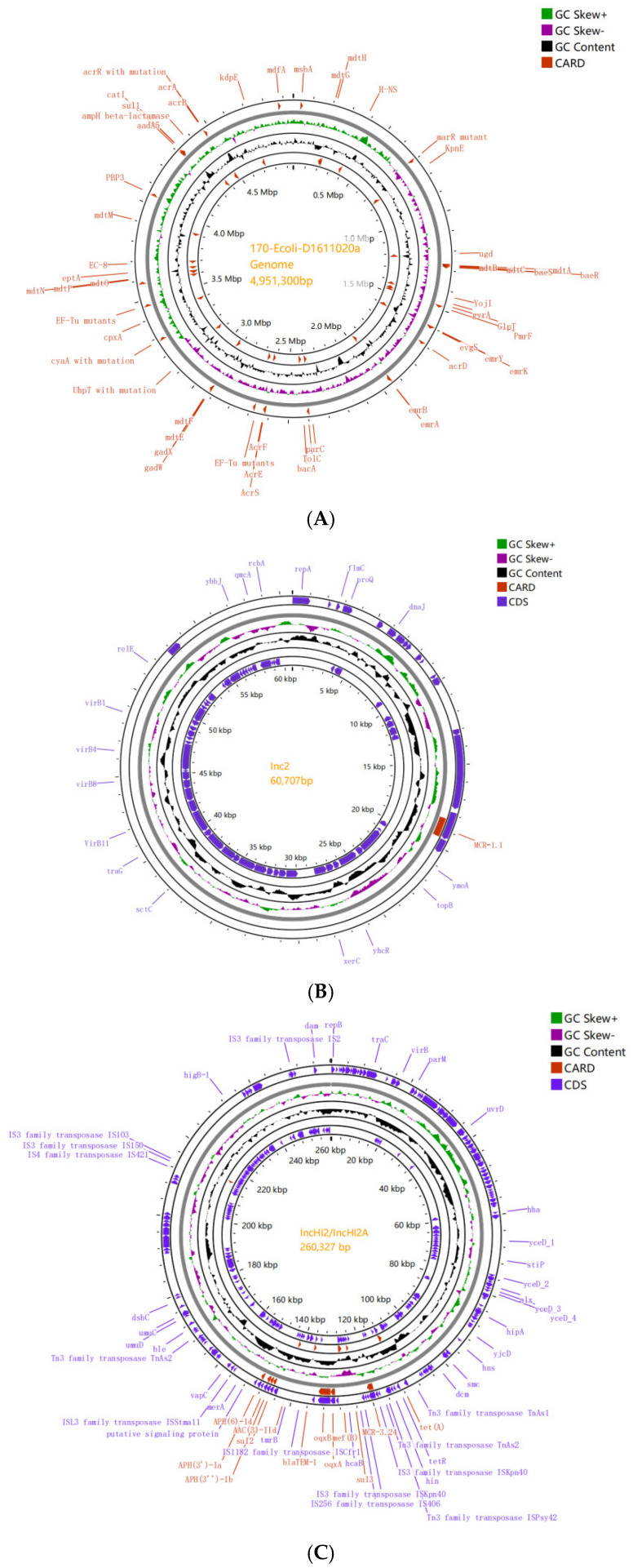
Chromosome and plasmid environments in the *mcr-1.1*/*mcr-3.24*-coharboring *E. coli* strain (170-Ecoli-A1611020a). (**A**) Chromosome size was 4.9 Mb. (**B**) *mcr-1.1* was contained in the plasmid Incl2 (60.7 kb) without other resistant genes. (**C**) *mcr-3.24* gene was located on plasmid IncHI2/IncHI2A with resistance genes *tet(A)*, *bla_TEM-1B_*, *aac(3)-IId*, *oqxB*, *sul3*, *bleO*, *oqxA*, and *mef(B)* and virulence gene *terC*.

By sequencing, we obtained a chromosome with a size of 4.9 Mb (Figure 9A) and seven circular plasmids in 170-Ecoli-A1611020a (IncI2, IncHI2/IncHI2A, IncFII, IncFIC(FII), IncFIA/IncFII/IncFIB, Col440I, and p011), with sizes ranging from 3.0 kb to 260.3 kb.

*mcr-1.1* was contained in plasmid Incl2 (60.7 kb) without any other resistant genes or IS upstream or downstream. The *mcr-1.1* gene (identity 100% to accession KP347127) was located on plasmid IncI2 (located on 17.8–19.5 kb) (Figure 9B). *mcr-3.24* with upstream and downstream ISKpn40 and sul3 with downstream IS406 were contained in plasmid IncHI2/IncHI2A (260.3 kb). *mcr-3.24* gene (identity 100% to accession NG060580) was located on plasmid IncHI2/IncHI2A (located on 119.0–120.7 kb) with eight resistance genes (*tet(A), bla_TEM-1B_*, *aac(3)-IId*, *oqxB*, *sul3*, *bleO*, *oqxA*, and *mef(B)*) and virulence gene *terC.* The *tet(A)* and *tet(R)* genes were contained alongside the Tn3 family transposase upstream and downstream in the plasmid IncHI2/IncHI2A (Figure 9C, Supplementary Figure S1).

Eight resistance genes (*APH(3′)-Ia*, *mphA*, *sul1*, *qacEΔ1*, *aadA16*, *dfrA27*, *arr-3*, and *tetM*) were contained in plasmid IncFII (102.3 kb) with *IS1595* family transposase *ISSsu9*, Tn3 family transposase *ISKox2*, and *ISEc63*. Eight resistance genes (*tet(B)*, *bla_OXA-1_*, *aadA5*, *sul1*, *APH(3′′)-Ib*, *APH(6)-Id*, *mphA*, and *ErmB*) were contained in plasmid (107.8 kb) exhibited the IncFIA/IncFII/IncFIB compound with the *IS1* family transposase *IS1R*, *IS6* family transposase *IS26*, *IS21* family transposase *ISPkr1*, and Tn3 family transposase. No resistance and virulence genes were found in plasmids p011 (101.1 kb), IncFIC (FII) (90.1 kb), and Col440I (3.0 kb).

### 2.6. Genetic Background Analysis of mcr-1.1- and bla_NDM-5_-Coharboring E. coli Strains

To understand the genetic background of *mcr-1.1-* and *bla_NDM-5_*-coharboring *E. coli* strains, two *E. coli* strains (29-Ecoli-D1903006c and 144-Ecoli-D1903012a) coharboring the *mcr-1.1* and *bla_NDM-5_* genes were selected for long-read complete sequencing. The sequencing result of 29-Ecoli-D1903006c is the same as 144-Ecoli-D1903012a with ST 2973; therefore, we choose 29-Ecoli-D1903006c to illustrate the genetic background.

**Figure 10:**
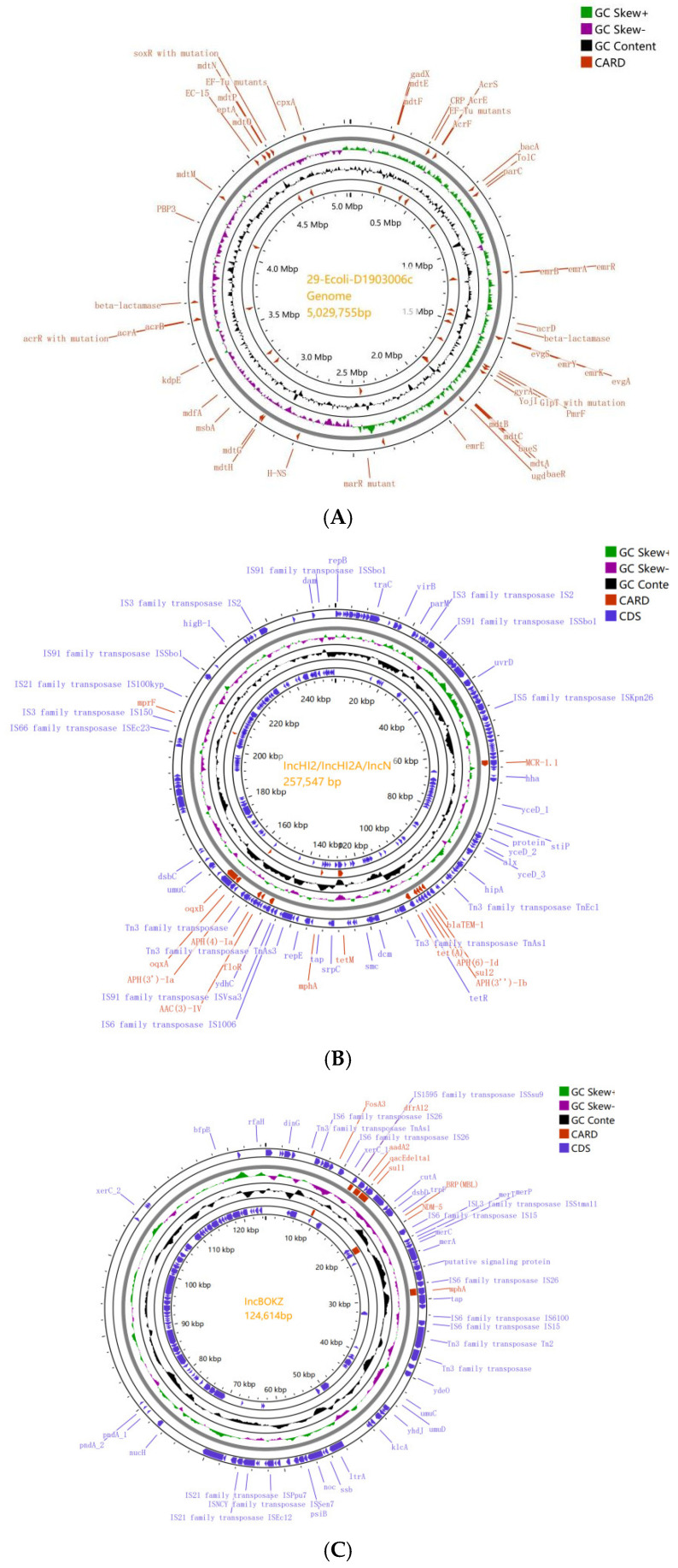
Chromosome and plasmid environments in *mcr-1.1*/*bla_NDM_*-coharboring *E. coli* strain(29-Ecoli-D1903006c). (**A**) Chromosome size was 5029 kb. (**B**) The *mcr-1.1* gene was located on plasmid IncHI2/IncHI2A/IncN (257.5 kb), with resistance genes *bla_TEM-1B_*, *sul2*, *aph(3′′)-Ib*, *aph(6)-Id*, *tet(A)*, *tet(M)*, *mph(A)*, *floR*, *aac(3)-IV*, *aph(4)-Ia*, *aph(3′)-Ia*, *oqxA*, *oqxB*, and *mprF* and virulence gene *terC*. (**C**) *bla_NDM-5_* was located on plasmid IncB/O/K/Z (124.6 kb) with resistance genes *fosA3*, *dfrA12*, *aadA2*, *qacEΔ1*, *sul1 and mph(A)* and virulence gene *traT*.

By sequencing, we obtained the chromosome with a size of 5,029 kb (Figure 10A) and four circular plasmids in 29-Ecoli-D1903006c (IncHI2/IncHI2A/IncN, IncBOKZ, IncI/IncFIB(AP001918), and Col440I), with sizes ranging from 2.1 kb to 257.5 kb.

The analysis of 29-Ecoli-D1903006c determined that the *mcr-1.1* gene (identity 100% to accession KP347127) was located in 62.1–63.7 kb on plasmid IncHI2/IncHI2A/IncN (257.5 kb), with eleven resistance genes (*bla_TEM-1B_*, *sul2*, *aph(3′’)-Ib*, *aph(6)-Id*, *tet(A)*, *tet(M)*, *mph(A)*, *floR*, *aac(3)-IV*, *aph(4)-Ia*, *aph(3′)-Ia*, *oqxA*, *oqxB*, and *mprF*) and virulence gene *terC. bla_NDM-5_* with downstream *IS6* family transposase *IS15* was contained in plasmid IncB/O/K/Z (124.6 kb) (Figure 10B, Supplementary Figure S2). *bla_NDM-5_* gene (identity 100% to accession JN104597) was located on plasmid IncB/O/K/Z (located on 19.6–20.4 kb) with six resistance genes (*fosA3*, *dfrA12*, *aadA2*, *qacEΔ1*, *sul1*, *and mph(A)*) and virulence gene *traT* (Figure 10C, Supplementary Figure S2). No resistance and virulence genes were found in plasmids IncI/IncFIB(AP001918) (162.7 kb) and Col440I (2.1 kb).

### 2.7. Genetic Background Analysis of tet(X4)-Harboring E. coli Strains

**Figure 11:**
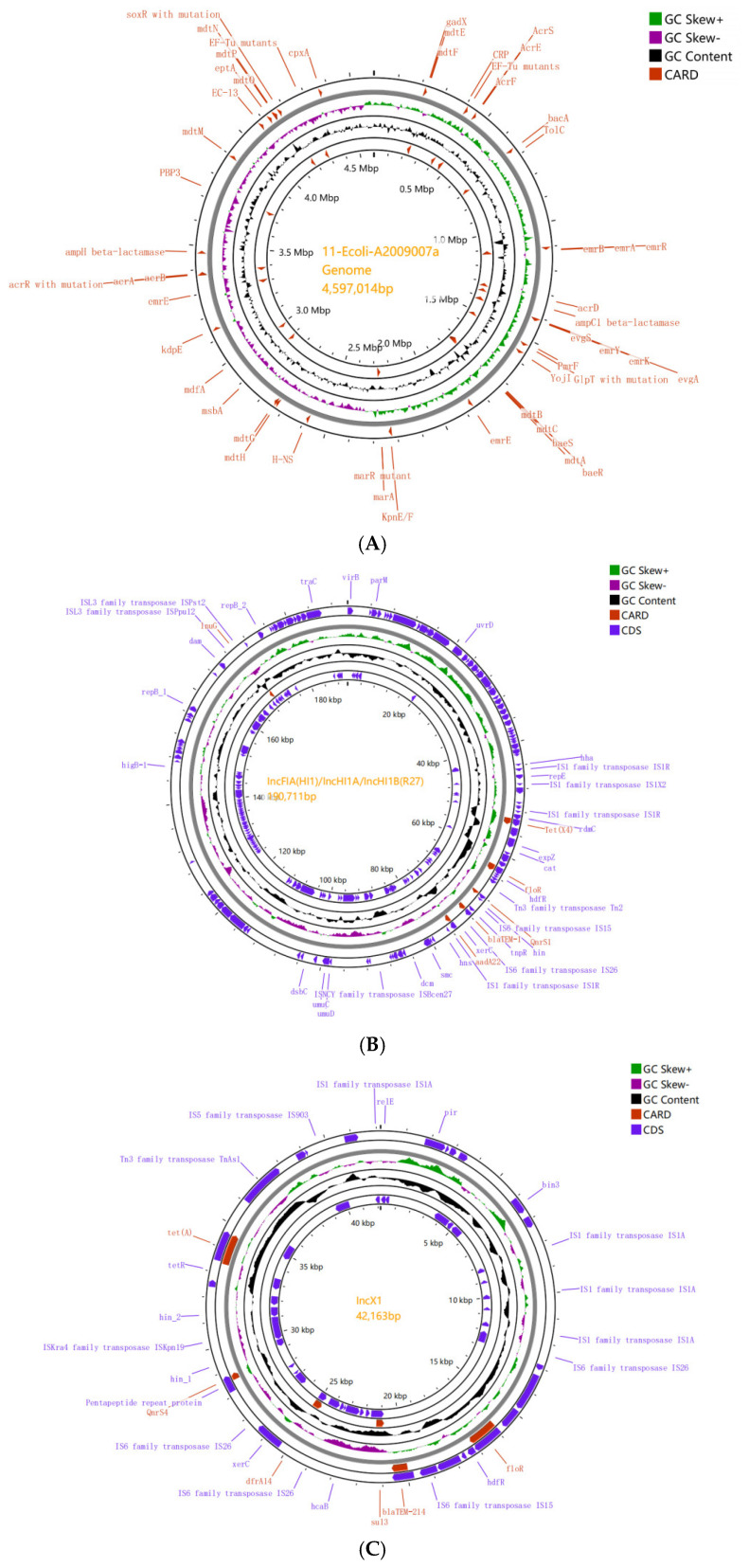
Chromosome and plasmid environments in the *Tet(X4)*-positive *E. coli* strain (11-Ecoli-A2009007a). (**A**) Chromosome size was 4597 kb. (**B**) *Tet(X4)* gene (identity 100% to accession MK134376) located on plasmid IncFIA(HI1)/IncHI1A/IncHI1B(R27) (located on 53.3–54.5 kb) with five resistance genes (*floR*, *qnrS1*, *bla_TEM-1B_*, *aadA22*, and *lnu(G)*). (**C**) *FloR*, *bla_TEM-214_*, *sul3*, *dfrA14*, *qnrS4*, and *tet(A)* were found in plasmid IncX1.

To understand the genetic background of *tet(X4)*-carrying *E. coli* strains, isolate 11-Ecoli-A2009007a with ST1720 was selected for long-read complete sequencing. By sequencing, we obtained the chromosome with a size of 4597 kb and two circular plasmids in 11-Ecoli-A2009007a (IncFIA(HI1)/IncHI1A/IncHI1B(R27) and IncX1) with sizes 190.7 kb and 42.1 kb, respectively (Figure 11A).

The analysis of 11-Ecoli-A2009007a determined that the *tet(X4)* gene (identity 100% to accession MK134376) was located on plasmid IncFIA(HI1)/IncHI1A/IncHI1B(R27) (located on 53.3–54.5 kb) with five resistance genes (*floR*, *qnrS1*, *bla_TEM-1B_*, *aadA22*, and *lnu(G)*) (Figure 11B, Supplementary Figure S3). Six resistance genes (*floR*, *bla_TEM-214_*, *sul3*, *dfrA14*, *qnrS4*, and *tet(A)*) were found in plasmid IncX1 (Figure 11C).

### 2.8. Statistics of Correlation Analysis Between mcr and the Related Resistance Factors

**Figure 12:**
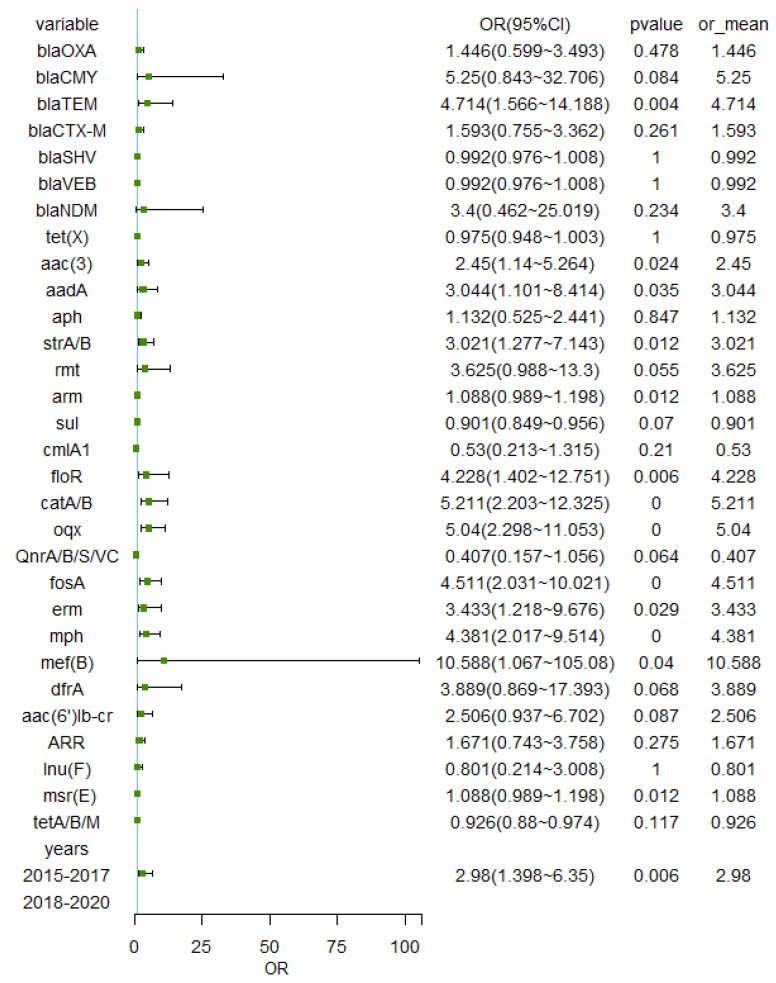
Forest plot of the OR characters to show the relation of the overall factors with *mcr* genes. OR: odds ratio. CI: confidence interval. The square in the forest plot represents the OR value, and the horizontal line represents the 95% CI.

**Figure 13:**
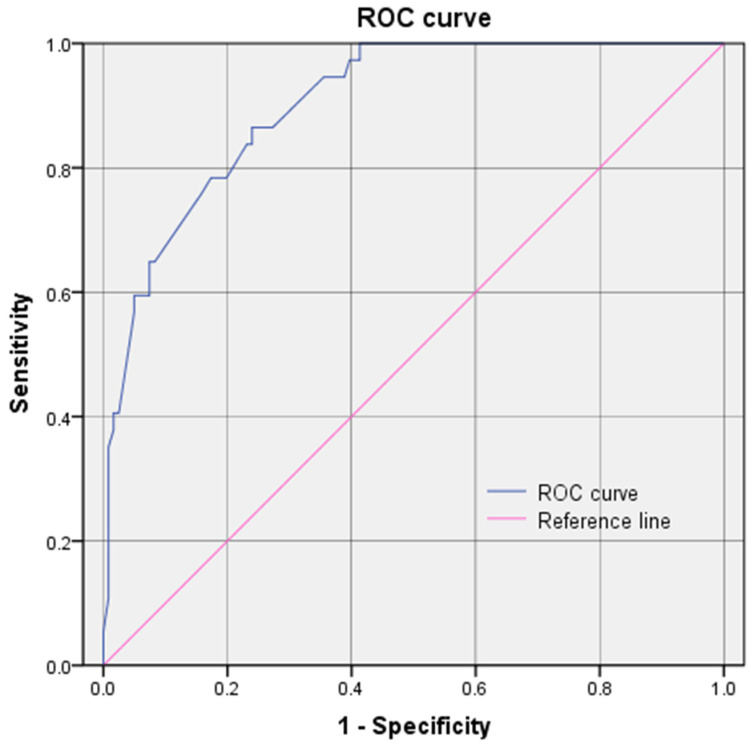
The ROC curve for the baseline model from the study showed an overall AUC of 0.903 (lower limit of 0.854 to an upper limit of 0.952) with a 95% confidence band.

To understand the correlation between resistance genes, binary logistic regression analysis and bivariable analysis were used to co-analyze their relationship with ARGS. Twelve factors (*bla_TEM_*, *aac(3)*, *aadA*, *strA/B*, *floR*, *catA/B*, *oqx*, *fosA*, *erm*, *mph*, *mef(B)*, and years (2015–2017)) related to *mcr* genes were screened from thirty two factors using Fisher’s exact test (*p* value < 0.05 and OR value with a 95% confidence interval >1 or < 1) (Figure 12). And seven risk factors (*bla_TEM_*, strA/B, *floR*, *catA/B*, years, *mph*, *and aadA)* were confirmed (*p* < 0.05) to create the logistic model followed by binary logistic regression analysis (backward elimination). The ROC curve for the baseline model from the study showed an overall AUC of 0.903 (lower limit of 0.854 to an upper limit of 0.952) with a 95% confidence band (Figure 13). ROC = 2.848 × X1 + 2.928 × X2 + 2.030 × X3 + 2.488 × X4 + 1.326 × X5 + 1.208 × X6 + 1.550 × X7 − 9.984 (X1: *floR*; X2: *catA/B*; X3: *bla_TEM_*; X4: Years (2015–2017); X5: *strA/B*; X6: *mph*; X7: *aadA*).

**Figure 14:**
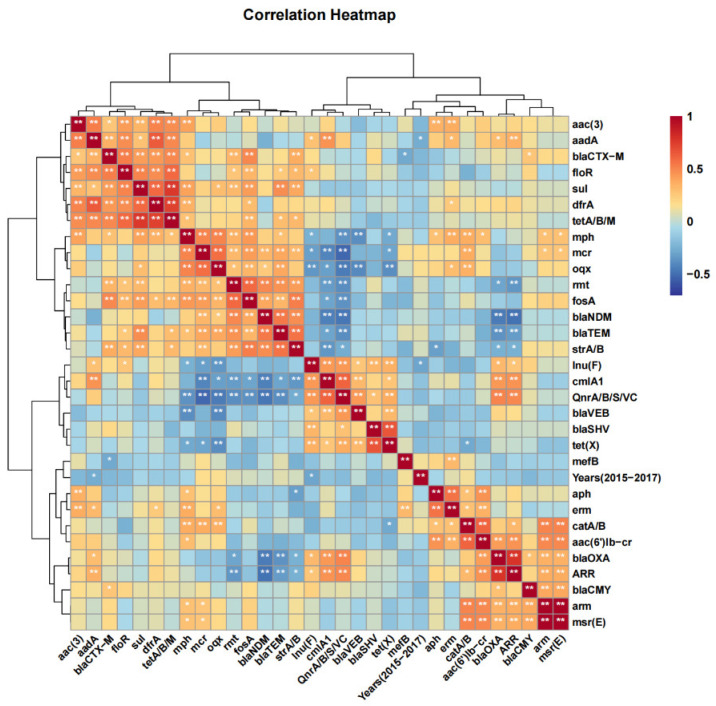
Heatmap and cluster analysis for correlation between resistance gene-related factors using the Euclidean and Ward.D of hcluster (hierarchical clustering) method based on Kendall (positive correlation threshold > 0.5, negative correlation threshold < −0.5, and threshold of *p* value < 0.01) (* *p* < 0.05; ** *p* < 0.01).

Meanwhile, bivariable and cluster network analyses for the correlation between overall resistance genes were conducted using Euclidean and Ward.D of hcluster (Hierarchical clustering method) methods based on Kendall (positive correlation threshold > 0.5, negative correlation threshold < 0.5, and threshold of *p* value < 0.01). The results of the bivariable analysis showed that six factors (*oqx*, *mph*, *fosA*, *bla_TEM_*, *catA/B*, *and strA/B*) related to *mcr* genes were consistent with the factors which were screened using Fisher’s exact test (Figure 14).

**Figure 15:**
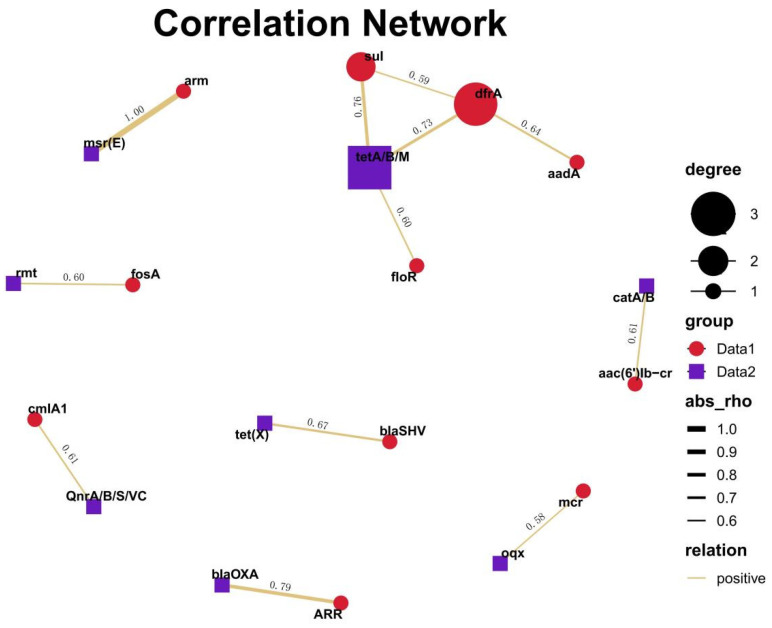
Cluster network analysis for the correlation between resistance genes. The data were screened using a positive correlation threshold > 0.58, a negative correlation threshold < −0.58, and a *p* value threshold of < 0.05. Data 1 and data 2 of the group are the resistance genes used for the pairwise correlation analysis.

And then, eleven factors (*oqx*, *QnrA/B/S/VC*, *mph*, *fosA*, *cmlA1*, *bla_TEM_*, *catA/B*, *bla_NDM_*, *rmt*, *strA/B*, *and lnu(F)*) related to *mcr* genes were screened using a positive correlation threshold > 0.58, a negative correlation threshold < −0.58, and a *p* value threshold of < 0.05. The results of the correlation network showed that positive correlations existed between *mcr* and *oqx*, for example. Additionally, apart from the correlation between *mcr* genes with other factors, some other correlations were revealed. The results of the correlation network based on the bivariable analysis showed that positive correlations existed between, for example, ARR and *bla_OXA_*, *bla_SHV_* and *tet(X)*, dfrA and *tetA/B/M*, *sul* and *tetA/B/M*, *aadA* and *drfA*, *arm* and *msr(E)*, etc. (Figure 15).

## 3. Materials and Methods

### 3.1. Isolation and Identification of E. coli Isolates

A total of 158 *E. coli* isolates were isolated from the livers and brains of diseased or dead livestock and poultry received from veterinary clinics in Shandong Province during 2015–2020. These diseased or dead livestock and poultry samples were taken from different farms. Liver and brain samples of livestock and poultry were collected aseptically to reduce microbial contamination. After grinding the samples with PBS, the supernatant was taken by centrifugation and cultured in Orientation Chromogenic Medium (CHROMagar™, Paris, France) overnight at 37 °C. All of the strains were identified using 16S rRNA sequence alignment (Novogene Co. Ltd., Tianjin, China).

### 3.2. Antimicrobial Susceptibility Testing

The minimum inhibitory concentrations (MICs) of ceftazidime, cefotaxime, meropenem, colistin, tigecycline, amikacin, amoxicillin clavulanate, and ciprofloxacin were determined using the broth microdilution method and the results were interpreted in accordance with the European Committee on Antimicrobial Susceptibility Testing (EUCAST) breakpoints (Version 12.0, valid from 1 January 2022) (http://www.eucast.org (accessed on 12 March 2020)). *E. coli* ATCC 25922 was used as a quality control strain. According to the EUCAST standards, we defined *E. coli* with different antibiotic resistance, such as ESBL-EC (extended-spectrum beta-lactamase-producing *E. coli*) as non-susceptible to cefotaxime (MIC > 1 mg/L) and susceptible to ertapenem (MIC ≤ 0.5 mg/L); CREC (carbapenem-resistant *E. coli*)s as non-susceptible to meropenem (MIC > 2 mg/L); COEC (colistin-resistant *E. coli*)s as non-susceptible to colistin (MIC > 2 mg/L); and TREC (tigecycline-resistant *E. coli*) as non-susceptible to tigecycline (MIC > 0.5 mg/L).

### 3.3. Whole-Genome Sequencing on an Illumina Platform and Assembly

Genomic DNA was extracted using Roche MagNA Pure 96. All of the *E. coli* strains were tested using second-generation high-throughput sequencing technology based on the Illumina HiSeq nova 6000 platform with 150 bp paired-end reads (Novogene Co. Ltd, Tianjin, China). The Illumina PCR adapter’s reads and the low-quality reads from the paired end were filtered through the quality control step using our own compiling pipeline. All good quality paired reads were assembled using SOAP denovo (https://sourceforge.net/projects/soapdenovo2/) (accessed on 16 May 2020)) [11,12], SPAdes [13] (https://openebench.bsc.es/tool/spades (accessed on 29 May 2020)), and ABySS [14] (http://www.bcgsc.ca/platform/bioinfo/software/abyss (accessed on 10 July 2020)) into a number of scaffolds. Then, the filter reads were handled by the next step of the gap-closing step.

### 3.4. Selected Strains for Long-Read Complete Sequencing

Isolates carrying *bla_NDM_*-, *mcr*-, and *tetX*-related genes were selected to identify the mobile genetic elements (MGE) and their relation to antimicrobial resistant genes and virulence factors. Whole-genome annotation was performed using RAST (Rapid Annotation using Subsystem Technology) (https://rast.nmpdr.org/ (accessed on 10 February 2021)). Circular plasmid maps were drawn, and plasmids were compared using Proksee (https://proksee.ca/projects (accessed on 8 March 2021)) and BLAST Ring Image Generator (BRIG v.0.95).

The de novo assembled contigs were MLST (7-gene Achtman ST scheme, whole-genome MLST, core-genome MLST, and ribosomal MLST) and serotyped in silico using EnteroBase typing tools [15]. The clean data were also analyzed using the following CGE databases, SerotypeFinder, MLSTtyper, PlasmidFinder, ResFinder, and VirulenceFinder [16,17,18,19,20], and the databases In Silico Clermont Phylotyper (https://ezclermont.hutton.ac.uk/ (accessed on 15 May 2021)). For the genomic relatedness comparison, we used different approaches based on the cgMLST of EnteroBase. Thus, a MLST tree was inferred using the MSTree V2 algorithm and the asymmetric distance matrix based on the cgMLST scheme from EnteroBase.

### 3.5. Single-Nucleotide Polymorphisms (SNPs) and Phylogeny Inference Analysis

CSI Phylogeny calls SNPs, filters the SNPs, conducts site validation, and infers phylogeny based on the concatenated alignment of the high-quality* SNPs. Whole-genome SNP analysis was performed using CSI phylogeny 1.4 [21].

### 3.6. Statistical Analysis

Statistical analyses were conducted by Fisher’s exact test of variance, as well as using a bivariable analysis at a significance level of *p* < 0.05. The correlation analysis between ARGs, times, and *mcr* characteristics and the ROC curve were performed using binary logistic regression analysis (backward elimination) by SPSS software (Version 22, IBM, New York, USA). The heatmap and cluster analysis for the correlation of ARGs and other factors was performed using the Euclidean and Ward.D of hcluster (hierarchical clustering) method [22] based on Kendall (positive correlation threshold > 0.5, negative correlation threshold < 0.5, and threshold of *p* value < 0.01) using R Version 3.6.3. The network analysis was carried out using R Version 3.6.3 and igraph crack 1.2.6.

## 4. Discussion

The data in this study showed that the sequence type (ST) diversity and resistance rate of poultry-derived *E. coli* from clinical cases was higher than that of livestock-derived ones. Notably, the positive rates of *E. coli* carrying the *mcr-1* in poultry sources were higher than those in livestock sources in each year from 2015 to 2020. To some extent, these indicates that the genetic diversity and severity of the drug resistance situation of pathogenic *E. coli* prevalent in Shandong Province during period 2015–2020 was higher in poultry sources than in livestock sources. In addition, about two-thirds of those clinical *E. coli* strains belong to phylogenetic group B1 and A. Generally, the commensal *E. coli* strains that survive in the intestines generally belong to groups A or B1 [23]. *E. coli* phylogenetic group B1 strains are known to be environmental strains, commonly occurring in different household animal species and freshwater beaches [24,25,26,27]. This indicates the possibility of the *E. coli* infection of livestock and poultry from feces and the environment.

The Chinese government’s ban of colistin as a growth promoter is effective. From our study, *mcr*-positive *E. coli* has displayed a positive relationship with years (2015–2017). China’s Ministry of Agriculture has issued notice No. 2428, dictating that colistin sulfate is no longer allowed to be added to feed as a growth promoter, as of 30 April 2017, and the relative abundance of *mcr*-1 in pig and chicken farms was lower in 2018 than 2017 in China [28]. However, the co-existence and co-transfer of resistance genes in livestock and poultry need more attention. It is common for one bacterial isolate to harbor a single *mcr* determinant. However, recent studies have revealed the co-existence of the *mcr*-1 and other *mcr* genes in *E. coli*, and *mcr*-1/*mcr*-3 is the most frequently detected combination of co-existing *mcr* genes in different countries [29]. Recent studies have also revealed the co-existence of *mcr*-1 and *bla_NDM_* genes in *E. coli* strains taken from chickens [30], hospital wastewater, and patient [31]. Further molecular surveillance of colistin resistance may reveal more combinations of co-existing *mcr* and *bla_NDM_* genes, and their influence on public health requires further assessment. From our study, *mcr*-positive *E. coli* has demonstrated a unique relationship with ARGS *bla_TEM_*, *floR*, *catA/B*, and *oqx*. It has been reported that *mcr*-1-positive *E. coli* could carry *oqx* with the proportion of 62.5% (10/16) in China in 2017 [32]. The co-transfer of *mcr-1.1*/*bla_TEM_*_-1B_ was observed to be located on different plasmids in Thailand in 2021 [33]. The co-occurrence of *mcr*-1 and *bla_TEM_* was observed in Tunisia in 2020 [34]. These are consistent with our data and further indicate the potential correlation of the co-existence of *bla_TEM_*, *floR*, *oqx*, and *mcr*. Although there is no special report on the correlation between *floR*, *catA/B*, and *mcr*, the potential correlation between them should not be ignored, and this highlights the impact of the overuse of both β-lactams and colistin in livestock and poultry production in China. It is also suggested that *bla_TEM_*-EC might be more likely to recruit the *mcr* gene than any other non-*bla_TEM_*-EC.

In addition, we found all four of the phylogroup F strains in the 37 *mcr*-positive *E. coli*, of which 2 were *mcr-1.1*-harboring strains with ST-354 and ST-117 from poultry and 2 were *mcr-1.1*/3.24-coharboring strains with ST-501 from livestock. It has been reported that chicken-source phylogroup F *E. coli* might pose a zoonotic risk and might contribute to the spread of MDR *E. coli* to humans [35]. This has been revealed as a true APEC that hold virulence [36]. As reported, phylogroup F *E. coli* strains from human and avian sources hold a higher content of ExPEC-related virulence genes and pathogenicity islands compared to those in the remaining new D and E groups [37]. ExPEC strains within phylogroup F, are also highly prevalent in companion animals, swine, horses, cattle, and wild birds [38,39,40,41]. Additionally, the two strains of bacteria co-carrying *mcr-1.1/3.24* isolated in this experiment have their *mcr-1.1* and *mcr-3.24* located on the Incl2 replicon plasmid and IncHI2/IncHI2A replicon plasmid, respectively. Existing reports show that, the Incl2 plasmid was prevalent in MCRPEC isolates in some areas of China [42,43]. The IncHI2/IncHI2A plasmid could be harboring *mcr* genes in MCRPEC, colistin-carbapenem-resistant *K. pneumoniae*, and *Salmonella Typhimurium* isolates [44,45,46]. In addition, there are transposons both upstream and downstream of them. Besides *mcr-1.1/3.24* strains, we also found two strains of avian-derived *E. coli* co-carrying *mcr1.1/bla_NDM_/fosA3* and three strains of porcine-derived *E. coli* co-carrying *tetx4/floR*, */qnrS1*. Carbapenems and tigecycline are the last-line drugs for treating *ESBL*-producing *E. coli*. Once *E. coli* becomes resistant to these two drugs, livestock and poultry will be at risk of having no drugs available for treatment. Livestock and poultry are important food sources and important sources of the transmission of antibiotic resistance genes for human beings. Therefore, the co-existence or co-transfer of resistance genes in livestock and poultry renders the situation of bacterial resistance even more serious, posing the risk of zoonosis and the transmission of super-resistant genes, and the spread of co-carriers such as *mcr-1.1*/3.24, *mcr1.1/bla_NDM_/fosA3*, and *tetx4/floR/qnrS1*.

## 5. Conclusions

This is the first report of *E. coli* strains (ST-501) from chickens coharboring a *mcr-1.1*-carrying Incl2 plasmid and a *mcr-3.24*-carrying IncHI2/IncHI2A plasmid, and the first report of *E. coli* strains (ST-2973) coharboring a *mcr-1.1*-carrying IncHI2A/IncHI2/IncN plasmid and a NDM-5-carrying IncB/O/K/Z plasmid in China. Our study also revealed the characterization of *Tet(X4)*-positive *E. coli* strains (ST-1720, ST-10, and ST-2509) contained in clinical pig samples from 2020 in Shandong province East China. We assessed the risk of animal-borne *Tet(X4)*-bearing *E. coli* to public health and the risk of zoonosis and the transmission of *mcr* genes, and the risk of the *mcr-1.1/3.24* co-carrier spread. The evolution and mechanism of *mcr* gene co-existence need further study to assess this gene’s impact on public health. Our data provide insight into the relationship of the co-existence and co-transfer between *mcr* and ARGS. Furthermore, the co-existence of different *mcr* and *bla_NDM_* genes in the same isolate presents a great challenge for infection control in MDR Gram-negative organisms. In light of reports of clone ST-501 belonging to high-virulent *E. coli*, programs to monitor this bacterium are urgently required to avoid its spread and zoonotic transmission to humans [47]. Therefore, there is an urgent need for further surveillance as well as the development of effective control measures to preserve the potency of these essential antibiotics.

## Data Availability

The original contributions presented in the study are included in the article/Supplementary Material; further inquiries can be directed to the corresponding author(s).

## Supplementary Materials

The following supporting information can be downloaded at: https://www.mdpi.com/article/10.3390/antibiotics14010095/s1 Figure S1: *mcr-3.24* gene was located on plasmid IncHI2/IncHI2A; Figure S2: The *mcr-1.1* gene was located on plasmid IncHI2/IncHI2A/IncN (257.5 kb) and *bla_NDM-5_* was located on plasmid IncB/O/K/Z (124.6 kb). Figure S3: *Tet(X4)* gene (identity 100% to accession MK134376) located on plasmid IncFIA(HI1)/IncHI1A/IncHI1B(R27).

## References

[1] Kromann S., Olsen R.H., Bojesen A.M., Jensen H.E., Thøfner I. (2021). Protective Potential of an Autogenous Vaccine in an Aerogenous Model of *Escherichia coli* Infection in Broiler Breeders. Vaccines.

[2] Johnson T.J., Kariyawasam S., Wannemuehler Y., Mangiamele P., Johnson S.J., Doetkott C., Skyberg J.A., Lynne A.M., Johnson J.R., Nolan L.K. (2007). The genome sequence of avian pathogenic *Escherichia coli* strain O1:K1:H7 shares strong similarities with human extraintestinal pathogenic *E. coli* genomes. J. Bacteriol..

[3] Messele Y.E., Trott D.J., Hasoon M.F., Veltman T., McMeniman J.P., Kidd S.P., Djordjevic S.P., Petrovski K.R., Low W.Y. (2023). Phylogenetic Analysis of *Escherichia coli* Isolated from Australian Feedlot Cattle in Comparison to Pig Faecal and Poultry/Human Extraintestinal Isolates. Antibiotics.

[4] Jakobsen L., Kurbasic A., Skjøt-Rasmussen L., Ejrnaes K., Porsbo L.J., Pedersen K., Jensen L.B., Emborg H.D., Agersø Y., Olsen K.E. (2010). *Escherichia coli* isolates from broiler chicken meat, broiler chickens, pork, and pigs share phylogroups and antimicrobial resistance with community-dwelling humans and patients with urinary tract infection. Foodborne Pathog. Dis..

[5] Overdevest I., Willemsen I., Rijnsburger M., Eustace A., Xu L., Hawkey P., Heck M., Savelkoul P., Vandenbroucke-Grauls C., van der Zwaluw K. (2011). Extended-spectrum beta-lactamase genes of *Escherichia coli* in chicken meat and humans, The Netherlands. Emerg. Infect. Dis..

[6] Yong C., Shinji S., Nobuyuki S. (2018). Current epidemiology, genetic evolution and clinical impact of extended-spectrum β-lactamase-producing *Escherichia coli* and Klebsiella pneumoniae. Infect. Genet. Evol..

[7] Sun J., Zeng X., Li X.P., Liao X.P., Liu Y.H., Lin J. (2017). Plasmid-mediated colistin resistance in animals: Current status and future directions. Anim. Health Res. Rev..

[8] He T., Wang R., Liu D., Walsh T.R., Zhang R., Lv Y., Ke Y., Ji Q., Wei R., Liu Z. (2019). Emergence of plasmid-mediated high-level tigecycline resistance genes in animals and humans. Nat. Microbiol..

[9] Liu C., Wang P., Dai Y., Liu Y., Song Y., Yu L., Feng C., Liu M., Xie Z., Shang Y. (2020). Longitudinal monitoring of multidrug resistance in *Escherichia coli* on broiler chicken fattening farms in Shandong, China. Poult. Sci..

[10] Zhao X., Liu Z., Zhang Y., Yuan X., Hu M., Liu Y. (2020). Prevalence and Molecular Characteristics of Avian-Origin mcr-1-Harboring *Escherichia coli* in Shandong Province, China. Front. Microbiol..

[11] Chaisson M.J.P., Wilson R.K., Eichler E.E. (2015). Genetic variation and the de novo assembly of human genomes. Nat. Rev. Genet..

[12] Li R., Li Y., Kristiansen K., Wang J. (2008). SOAP: Short oligonucleotide alignment program. Bioinformatics.

[13] Bankevich A., Nurk S., Antipov D., Gurevich A.A., Dvorkin M., Kulikov A.S., Lesin V.M., Nikolenko S.I., Pham S., Prjibelski A.D. (2012). SPAdes: A new genome assembly algorithm and its applications to single-cell sequencing. J. Comput. Biol. J. Comput. Mol. Cell Biol..

[14] Simpson J.T., Wong K., Jackman S.D., Schein J.E., Jones S.J., Birol I. (2009). ABySS: A parallel assembler for short read sequence data. Genome Res..

[15] Alikhan N., Zhou Z., Sergeant M.J., Achtman M. (2018). A genomic overview of the population structure of Salmonella. PLoS Genet..

[16] Joensen K.G., Tetzschner A.M., Iguchi A., Aarestrup F.M., Scheutz F. (2015). Rapid and Easy In Silico Serotyping of *Escherichia coli* Isolates by Use of Whole-Genome Sequencing Data. J. Clin. Microbiol..

[17] Larsen M.V., Cosentino S., Rasmussen S., Friis C., Hasman H., Marvig R.L., Jelsbak L., Sicheritz-Pontén T., Ussery D.W., Aarestrup F.M. (2012). Multilocus sequence typing of total-genome-sequenced bacteria. J. Clin. Microbiol..

[18] Camacho C., Coulouris G., Avagyan V., Ma N., Papadopoulos J., Bealer K., Madden T.L. (2009). BLAST+: Architecture and applications. BMC Bioinform..

[19] Carattoli A., Zankari E., García-Fernández A., Voldby L.M., Lund O., Villa L., Møller A.F., Hasman H. (2014). In silico detection and typing of plasmids using PlasmidFinder and plasmid multilocus sequence typing. Antimicrob. Agents Chemother..

[20] Zankari E., Hasman H., Cosentino S., Vestergaard M., Rasmussen S., Lund O., Aarestrup F.M., Larsen M.V. (2012). Identification of acquired antimicrobial resistance genes. J. Antimicrob. Chemother..

[21] Kaas R.S., Leekitcharoenphon P., Aarestrup F.M., Lund O. (2014). Solving the problem of comparing whole bacterial genomes across different sequencing platforms. PLoS ONE.

[22] Fionn M., Pierre L. (2014). Ward’s Hierarchical Agglomerative Clustering Method: Which Algorithms Implement Ward’s Criterion?. J. Classif..

[23] Clermont O., Christenson J.K., Denamur E., Gordon D.M. (2013). The Clermont *Escherichia coli* phylo-typing method revisited: Improvement of specificity and detection of new phylo-groups. Environ. Microbiol. Rep..

[24] Walk S.T., Alm E.W., Calhoun L.M., Mladonicky J.M., Whittam T.S. (2007). Genetic diversity and population structure of *Escherichia coli* isolated from freshwater beaches. Environ. Microbiol..

[25] Katarzyna B., Paweł M., Michał S. (2008). Phylogenetic background, virulence gene profiles, and genomic diversity in commensal *Escherichia coli* isolated from ten mammal species living in one zoo. Vet. Microbiol..

[26] Ziebell K., Konczy P., Yong I., Frost S., Mascarenhas M., Kropinski A.M., Whittam T.S., Read S.C., Karmali M.A. (2008). Applicability of phylogenetic methods for characterizing the public health significance of verocytotoxin-producing *Escherichia coli* strains. Appl. Environ. Microbiol..

[27] Carlos C., Pires M.M., Stoppe N.C., Hachich E.M., Sato M.I., Gomes T.A., Amaral L.A., Ottoboni L.M. (2010). *Escherichia coli* phylogenetic group determination and its application in the identification of the major animal source of fecal contamination. BMC Microbiol..

[28] Wang Y., Pires M.M., Stoppe N.C., Hachich E.M., Sato M.I., Gomes T.A., Amaral L.A., Ottoboni L.M. (2020). Changes in colistin resistance and mcr-1 abundance in *Escherichia coli* of animal and human origins following the ban of colistin-positive additives in China: An epidemiological comparative study. Lancet Infect. Dis..

[29] Li R., Zhang P., Yang X., Wang Z., Fanning S., Wang J., Du P., Bai L. (2019). Identification of a novel hybrid plasmid coproducing MCR-1 and MCR-3 variant from an *Escherichia coli* strain. J. Antimicrob. Chemother..

[30] Liu B.T., Song F.J., Zou M., Zhang Q.D., Shan H. (2017). High Incidence of *Escherichia coli* Strains Coharboring mcr-1 and bla(NDM) from Chickens. Antimicrob. Agents Chemother..

[31] Zhu L., Shuai X.Y., Lin Z.J., Sun Y.J., Zhou Z.C., Meng L.X., Zhu Y.G., Chen H. (2022). Landscape of genes in hospital wastewater breaking through the defense line of last-resort antibiotics. Water Res..

[32] Zhou H.W., Zhang T., Ma J.H., Fang Y., Wang H.Y., Huang Z.X., Wang Y., Wu C., Chen G.X. (2017). Occurrence of Plasmid- and Chromosome-Carried mcr-1 in Waterborne Enterobacteriaceae in China. Antimicrob. Agents Chemother..

[33] Trongjit S., Chuanchuen R. (2021). Whole genome sequencing and characteristics of *Escherichia coli* with co-existence of ESBL and mcr genes from pigs. PLoS ONE.

[34] Sana D., Soufi L., Hamza A., Fedida D., Zied C., Awadhi E., Mtibaa M., Hassen B., Cherif A., Torres C. (2020). Co-occurrence of mcr-1 mediated colistin resistance and β-lactamase-encoding genes in multidrug-resistant *Escherichia coli* from broiler chickens with colibacillosis in Tunisia. J. Glob. Antimicrob. Resist..

[35] Wang M., Jiang M., Wang Z., Chen R., Zhuge X., Dai J. (2021). Characterization of antimicrobial resistance in chicken-source phylogroup F *Escherichia coli*: Similar populations and resistance spectrums between *E. coli* recovered from chicken colibacillosis tissues and retail raw meats in Eastern China. Poult. Sci..

[36] Zhuge X., Zhou Z., Jiang M., Wang Z., Sun Y., Tang F., Xue F., Ren J., Dai J. (2020). Chicken-source *Escherichia coli* within Phylogroup F Shares Virulence Genotypes and is Closely Related to Extraintestinal pathogenic *E. coli* (ExPEC) Causing Human Infections. Transbound. Emerg. Dis..

[37] Logue C.M., Wannemuehler Y., Nicholson B.A., Doetkott C., Barbieri N.L., Nolan L.K. (2017). Comparative Analysis of Phylogenetic Assignment of Human and Avian ExPEC and Fecal Commensal *Escherichia coli* Using the (Previous and Revised) Clermont Phylogenetic Typing Methods and its Impact on Avian Pathogenic *Escherichia coli* (APEC) Classification. Front. Microbiol..

[38] Ewers C., Bethe A., Stamm I., Grobbel M., Kopp P.A., Guerra B., Stubbe M., Doi Y., Zong Z., Kola A. (2014). CTX-M-15-D-ST648 *Escherichia coli* from companion animals and horses: Another pandemic clone combining multiresistance and extraintestinal virulence?. J. Antimicrob. Chemother..

[39] Sam A., Jordan D., Wong H.S., Johnson J.R., Toleman M.A., Wakeham D.L., Gordon D.M., Turnidge J.D., Mollinger J.L., Gibson J.S. (2015). First detection of extended-spectrum cephalosporin- and fluoroquinolone-resistant *Escherichia coli* in Australian food-producing animals. J. Glob. Antimicrob. Resist..

[40] Blyton M.D.J., Pi H., Vangchhia B., Abraham S., Trott D.J., Johnson J.R., Gordon D.M. (2015). Genetic Structure and Antimicrobial Resistance of *Escherichia coli* and Cryptic Clades in Birds with Diverse Human Associations. Appl. Environ. Microbiol..

[41] Guo S., Wakeham D., Brouwers H.J., Cobbold R.N., Abraham S., Mollinger J.L., Johnson J.R., Chapman T.A., Gordon D.M., Barrs V.R. (2015). Human-associated fluoroquinolone-resistant *Escherichia coli* clonal lineages, including ST354, isolated from canine feces and extraintestinal infections in Australia. Microbes Infect..

[42] Lin H., Chen W., Zhou R., Yang J., Wu Y., Zheng J., Fei S., Wu G., Sun Z., Li J. (2022). Characteristics of the plasmid-mediated colistin-resistance gene mcr-1 in *Escherichia coli* isolated from a veterinary hospital in Shanghai. Front. Microbiol..

[43] Ma J., Zhou W., Wu J., Liu X., Lin J., Ji X., Lin H., Wang J., Jiang H., Zhou Q. (2022). Large-Scale Studies on Antimicrobial Resistance and Molecular Characterization of *Escherichia coli* from Food Animals in Developed Areas of Eastern China. Microbiol. Spectr..

[44] Timmermans M., Wattiau P., Denis O., Boland C. (2021). Colistin resistance genes mcr-1 to mcr-5, including a case of triple occurrence (mcr-1, -3 and -5), in *Escherichia coli* isolates from faeces of healthy pigs, cattle and poultry in Belgium 2012–2016. Int. J. Antimicrob. Agents.

[45] Attalla E.T., Khalil A.M., Zakaria A.S., Baker D.J., Mohamed N.M. (2023). Genomic characterization of colistin-resistant Klebsiella pneumoniae isolated from intensive care unit patients in Egypt. Ann. Clin. Microbiol. Antimicrob..

[46] Diaconu E.L., Alba P., Feltrin F., Di Matteo P., Iurescia M., Chelli E., Donati V., Marani I., Giacomi A., Franco A. (2021). Emergence of IncHI2 Plasmids With Mobilized Colistin Resistance (mcr)-9 Gene in ESBL-Producing, Multidrug-Resistant Salmonella Typhimurium and Its Monophasic Variant ST34 From Food-Producing Animals in Italy. Front. Microbiol..

[47] Zhuge X., Jiang M., Tang F., Sun Y., Ji Y., Xue F., Ren J., Zhu W., Dai J. (2019). Avian-source mcr-1-positive *Escherichia coli* is phylogenetically diverse and shares virulence characteristics with *E. coli* causing human extra-intestinal infections. Vet. Microbiol..

