# Supplementary material for: Antimicrobial Resistance Genes in Clinical Escherichia coli Strains from Livestock and Poultry in Shandong Province, China During 2015–2020
Source: Antibiotics (Basel). 2025 Jan 15;14(1):95. doi: 10.3390/antibiotics14010095 (PMC11761920; doi:10.3390/antibiotics14010095)
Supplement: Supplementary file 1 [file antibiotics-14-00095-s001.zip › antibiotics-3406636-supplementary.pdf]

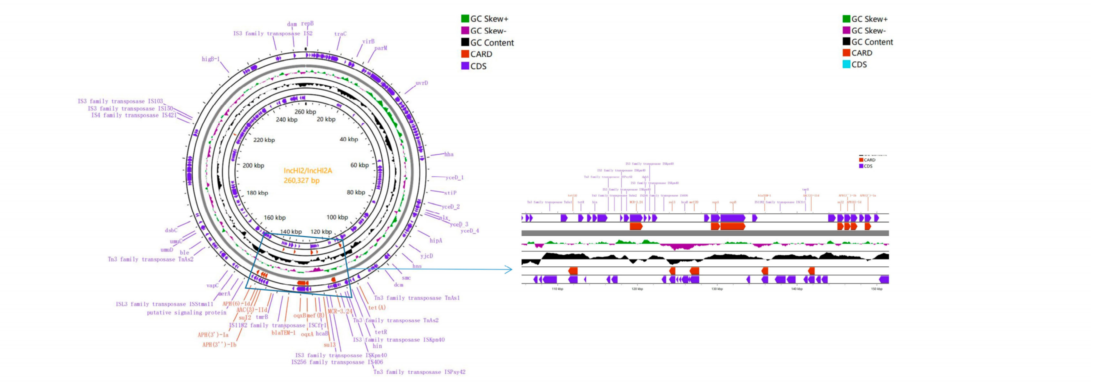

Figure S1. *mcr-3.24* gene was located on plasmid IncHI2/IncHI2A.

t

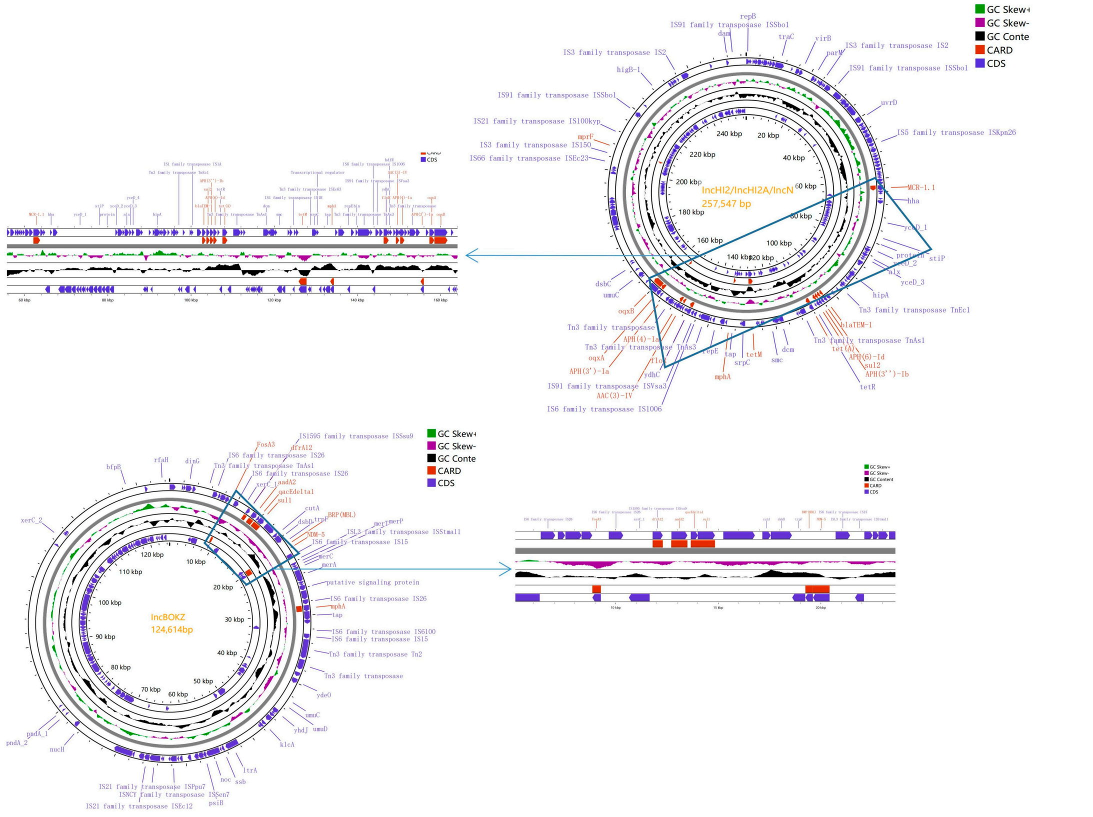

Figure S2. The *mcr-1.1* gene was located on plasmid IncHI2/IncHI2A/IncN (257.5 kb) and *bla*$_{NDM-5}$ was located on plasmid IncB/O/K/Z (124.6 kb).

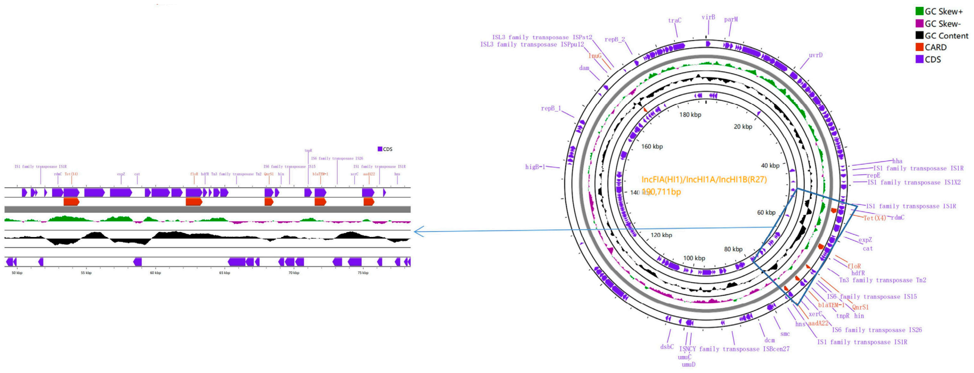

Figure S3. *Tet(X4)* gene (identity 100% to accession MK134376) located on plasmid IncFIA(HI1)/IncHI1A/IncHI1B(R27).
